# Evolutionary dynamics of mutants that modify population structure

**DOI:** 10.1098/rsif.2023.0355

**Published:** 2023-11-29

**Authors:** Josef Tkadlec, Kamran Kaveh, Krishnendu Chatterjee, Martin A. Nowak

**Affiliations:** ^1^ Department of Mathematics, Harvard University, Cambridge, MA 02138, USA; ^2^ Department of Organismic and Evolutionary Biology, Harvard University, Cambridge, MA 02138, USA; ^3^ Computer Science Institute, Charles University, Prague, Czech Republic; ^4^ Department of Applied Mathematics, University of Washington, Seattle, WA 98195, USA; ^5^ Institute of Science and Technology Austria, Am Campus 1, 3400 Klosterneuburg, Austria

**Keywords:** Moran process, evolutionary graph theory, fixation probability, spatial structure

## Abstract

Natural selection is usually studied between mutants that differ in reproductive rate, but are subject to the same population structure. Here we explore how natural selection acts on mutants that have the same reproductive rate, but different population structures. In our framework, population structure is given by a graph that specifies where offspring can disperse. The invading mutant disperses offspring on a different graph than the resident wild-type. We find that more densely connected dispersal graphs tend to increase the invader’s fixation probability, but the exact relationship between structure and fixation probability is subtle. We present three main results. First, we prove that if both invader and resident are on complete dispersal graphs, then removing a single edge in the invader’s dispersal graph reduces its fixation probability. Second, we show that for certain island models higher invader’s connectivity increases its fixation probability, but the magnitude of the effect depends on the exact layout of the connections. Third, we show that for lattices the effect of different connectivity is comparable to that of different fitness: for large population size, the invader’s fixation probability is either constant or exponentially small, depending on whether it is more or less connected than the resident.

## 1. Introduction

Evolutionary dynamics is the study of how different traits arise and disappear in a population of reproducing individuals. Each trait might confer a fitness advantage (or disadvantage) on its bearer, thus in turn altering the probability that the trait spreads through the population (an event called *fixation*) or disappears (*extinction*). Besides the fitness advantage, another important factor in determining the fate of a trait over time (its fixation or extinction) is the spatial structure of the population [1–5]. For instance, the population might be subdivided into ‘islands’: an offspring of a reproducing individual then typically stays in the same island, but occasionally it migrates to some nearby island. The fixation probability of a trait then crucially depends on the dispersal pattern, that is, the migration rates among the islands. Incorporation of population structure into a model of selection dynamics substantially improves the descriptive power of the model [1,5–11].

Evolutionary graph theory is a powerful framework for studying natural selection in population structures with arbitrarily complex dispersal patterns [12–19]. On an evolutionary graph (network), individuals occupy the nodes (vertices), and the edges (links) specify where the offspring can migrate. Graphs can represent spatial structures, contact networks in epidemiology, social networks, and phenotypic or genotypic structures in biological populations [12,20–24]. The question is then: how does a graph structure affect the fixation probability of a new mutant introduced into a background population of residents? Extensive research over the past decade has produced many remarkable population structures with various desirable properties [25–29]. As one example, consider a mutation that increases the reproduction rate of the affected individual. Population structures that increase the fixation probability of such mutations, as compared with the baseline case of unstructured (well-mixed) populations, are known as amplifiers of selection. Many amplifiers of selection are known [30–33].

In this work, we primarily consider mutations that do not change the reproductive rate of the affected individual, but rather its motility potential. That is, we consider mutants which perceive the population structure through a graph with additional edges (or with fewer edges). In nature, an altered motility potential could arise in a variety of scenarios. We give three examples.

First, consider a species occupying a region that is split by a geographical barrier into two parts. If the mutation allows the offspring to successfully cross the barrier, the mutants will perceive the population structure as being well-mixed, whereas the residents will continue perceiving it as being split into two parts (islands). This can be due to differences in migration potential between mutants and residents.

**Figure 1.**
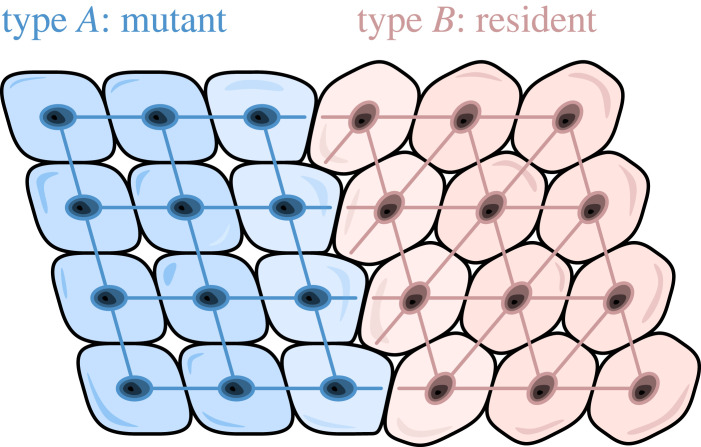
In epithelial tissues, different cell types align along different lattice-like structures.

As a second example, consider structured multicellular organisms. There, cells are arranged in symmetric lattice structures known as epithelia. An epithelial tissue may be described as a two-dimensional sheet defined by vertex points representing wall junctions, one-dimensional edges representing cell walls, and two-dimensional faces representing cells. The form of this tissue network is determined by the extracellular matrix (ECM). The ECM is a network consisting of extracellular macromolecules, collagen and enzymes that provide structural and biochemical support to surrounding cells. The composition of ECM varies between multicellular structures [34–38]. Thus, when discussing somatic evolution in multicellular organisms, the invading genotype might differ in what network structure it is forming [36,39]. In other words, each type, in the absence of the other type, forms its own and different extracellular matrix. This leads to different alignment of cells and thus a new population structure (figure 1).

Carcinoma is yet another example of how the tissue organization of the invader and resident types can differ from each other. In this case, tumour cells normally have a highly disorganized neighbourhood structure, due to the variability in cell–cell adhesion and the lack of proper epithelial programmes among tumour cells in the tumour microenvironment [40,41]. Normal epithelial cells, on the other hand, are typically organized in monolayers and form geometric lattice-like patterns [42]. Additionally, aggressive tumour cells, due to their high mesenchymal marker level, can show higher motility relative to normal tissue cells [43]. These two facts, namely the lack of normal epithelial structure in tumours and higher motility in aggressive cancers, can lead to substantial differences in invasion pattern and potentially affect the outcome of evolutionary processes.

In order to model differences in the motility potential within the framework of evolutionary graph theory, we represent the population structure as two graphs *G*^*A*^, *G*^*B*^ overlaid on top of each other on the same set of *N* nodes [44]. The *N* nodes represent different sites, each occupied by a single individual. The two graphs *G*^*A*^, *G*^*B*^ represent the dispersal patterns for the mutants and residents, respectively. In other words, mutant offspring migrate along the edges of *G*^*A*^, whereas resident offspring migrate along the edges of *G*^*B*^.

We study the fixation probability *ρ*(*G*^*A*^, *G*^*B*^) of a single neutral mutant which appears at a random node and perceives the population structure as *G*^*A*^, as it attempts to invade a population of residents which perceive the population through *G*^*B*^. We assume that both graphs *G*^*A*^ and *G*^*B*^ are connected. When the two structures *G*^*A*^ and *G*^*B*^ coincide, the fixation probability of a neutral mutant is equal to 1/*N*, regardless of the graph structure. By contrast, when the structures *G*^*A*^ and *G*^*B*^ differ, the fixation probability can be either higher or lower than 1/*N*.

There is a large body of the literature on the evolution and ecology of migration and dispersal [45–49], especially for population structures formed by islands (also called patches, demes or metapopulations) [50–53]. The present framework provides a formal way to approach the motility potential as a genotypic quality. Moreover, it enables a direct study of both simple and arbitrarily complex population structures, of any population size. In this way, the framework is a generalization of the vast literature on migration and dispersal, similarly to how evolutionary graph theory is a generalization of the vast literature on evolution and ecology in spatially structured populations [9,12].

Among the graph-theoretical approaches, other ways to model motility and dispersal have been suggested in the literature [54–56]. They allow for the offsprings to disperse in more complex forms and reach locations that are not directly connected to the mother location. This introduces migration potential as an independent quantity relative to the proliferation potential of the types [57–60]. In those cases, the motility potential is representative of a random motion, and it is typically decoupled from the reproduction events. For example, Manem *et al*. [58] considered a model where beside death–birth events, individual cell movements are also included. They also considered one cell type to have different motility potential than the other. It was observed that the fixation probability of the mutants is reduced when including individual cell movements. Such random motility and motion has an anti-synergistic relationship with the proliferation potential [55,57,58].

Here we show that, in contrast to random motility, enhanced structured motility generally leads to an increase in the fixation probability of the invading mutant. We do this by considering three types of population structures. First, we consider all 112 population structures on *N* = 6 nodes. By numerically calculating the relevant fixation probabilities, we show that increased motility potential is positively correlated with increased fixation probability. Moreover, we mathematically prove that for any population size *N* the Complete graph *K*_*N*_ is ‘locally optimal’ in a sense described in the next section. However, we note that the effect is subtle, and we identify specific circumstances in which making mutants more (structurally) motile actually decreases their fixation probability. Second, we consider large population structures corresponding to island models, and we show that the extent to which increased motility helps the mutant fixate can vary considerably, depending on the exact layout of the extra connections. Finally, we consider large low-dimensional lattices, and we show that the effect of altered structural motility is analogous to the effect of altered reproductive rate: in the limit of large population size, the fixation probability of a mutant is either constant or exponentially small, depending on whether it is more or less motile than the residents. This latter case of low-dimensional lattices is relevant for the study of somatic evolution in epithelial tissues and carcinoma.

## 2. Model

### 2.1. Standard Moran process on a graph

Within the framework of evolutionary graph theory [12], a population structure is described as a graph (network), where nodes (vertices) represent locations (sites) and the graph connectivity defines the topology and the neighbourhood. There are *N* nodes and each node is occupied by a single individual. Each individual is either of type *A* (mutant) with fitness *r*_*A*_, or of type *B* (resident) with fitness *r*_*B*_. The evolutionary dynamics is governed by the standard stochastic discrete-time Moran birth–death process, adapted to the population structure: at each time point, a single individual is picked for reproduction, proportionally to its fitness. This focal individual produces offspring (a copy of itself), and the offspring then migrates and replaces a random neighbouring individual.

The probability of migration from node *i* to node *j* is given by an *N* × *N* dispersal matrix $M={\left(m_{i,j}\right)}_{i,j=1}^{N}$. Thus, for undirected, unweighted graphs (which are the focus of this work), the entries *m*_*i*,*j*_ of the dispersal matrix *M* satisfy

$$m_{i,j}=\{\begin{array}{cc} \frac{1}{\deg(i)},\; & \mathrm{if}\;\mathrm{nodes}\;i\;\mathrm{and}\;j\;\mathrm{are}\;\mathrm{adjacent},\;\\ 0,\; & \mathrm{otherwise}.\; \end{array}$$

(Here deg⁡(u) is the *degree* of node *u*, that is, the number of nodes adjacent to *u*.)

### 2.2. Moran process on two graphs

**Figure 2.**
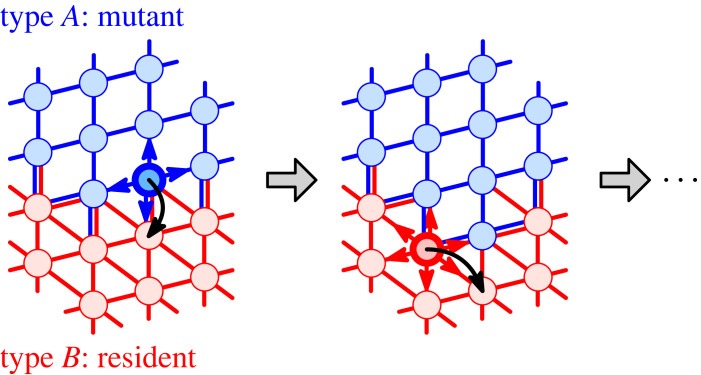
Moran process with type-dependent dispersal patterns. In each discrete time step, a random individual reproduces and the offspring proliferates to a neighbouring node. Type *A* offspring (mutant, blue) migrate along the edges of the blue graph *G*_*A*_, whereas type *B* offspring (residents, red) migrate along the red edges of *G*_*B*_. The key quantity is the fixation probability *ρ*(*G*^*A*^, *G*^*B*^) that a single initial mutant successfully invades the population of residents.

It is commonly assumed that the dispersal matrix is independent of the two types; that is, both types of individuals perceive the population through the same population structure. Following the recent work of Melissourgos *et al.* [44], here we study a more general case in which the dispersal pattern depends on the type of the offspring that migrates. Thus, we consider two graphs *G*^*A*^, *G*^*B*^ and the corresponding dispersal matrices $M^{A}={\left(m_{i,j}^{A}\right)}_{i,j=1}^{N}$, $M^{B}={\left(m_{i,j}^{B}\right)}_{i,j=1}^{N}$. That is, any time a type *A* individual reproduces at a node *i*, the offspring replaces an individual at node *j* with probability $m_{ij}^{A}$. By contrast, the offspring of a type *B* individual reproducing at node *i* migrates to node *j* with probability $m_{ij}^{B}$ (figure 2). We assume that both graphs *G*^*A*^ and *G*^*B*^ are connected.

The state of the population at any given time point is described by a list $n=(n_{1},\ldots ,n_{N})$ of *N* zeros and ones, where *n*_*i*_ = 1 denotes that node *i* is currently occupied by a type *A* individual (mutant). The model is a Markov chain with 2^*N*^ possible states. Two of the states are absorbing, and they correspond to homogeneous population consisting purely of type *A* individuals $\left(\mathrm{state}\;n^{1}=(1,\ldots ,1)\right)$ or type *B* individuals $\left(\mathrm{state}\;n^{0}=(0,\ldots ,0)\right)$.

### 2.3. Questions and results

In this work, we study how differences in the migration and dispersal pattern *G*^*A*^ of mutants and *G*^*B*^ of residents influence the fate of a single random mutant which appears at a random location. As a measure of the mutant success, we use its fixation probability under neutral drift (that is, *r*_*A*_ = *r*_*B*_). We denote this quantity by *ρ*(*G*^*A*^, *G*^*B*^). It is known that whenever the two types have the same dispersal pattern (*G*^*A*^ = *G*^*B*^), the fixation probability under neutral drift is equal to 1/*N*, regardless of the graph structure [61]. Thus, the regime of neutral drift provides a clean baseline and it decouples the effect of a difference in population structure from other effects. Specifically, we study the following questions:

(i) Does increased motility increase or decrease the mutant fixation probability?
(ii) Can the effect be quantified for simple, biologically plausible structures, such as island models or low-dimensional lattices?

To address the first question, in section Small graphs we numerically compute the fixation probabilities *ρ*(*G*^*A*^, *G*^*B*^) for all pairs *G*^*A*^, *G*^*B*^ of graphs of small size. We find that, generally speaking, increased motility potential (that is, living on a graph with more edges) tends to increase the fixation probability of the mutant. In particular, we prove that the Complete graph is ‘locally optimal’ in the sense that if residents live on a Complete graph and mutants live on a graph that misses a single edge, then the mutant fixation probability drops below the threshold 1/*N* (see theorem A.1 in appendix A). However, we also identify special cases, in which an increase in the motility potential decreases the fixation probability rather than increasing it. This suggests that for arbitrary population structures the effects of motility on the fixation probability are complex. Given this complexity, we proceed to study pairs of specific, biologically relevant structures.

Namely, in section Dense regular graphs we consider certain population structures that correspond to island models with two equal islands. We show that two such structures with the same total number of edges exhibit a substantially different behaviour in the limit *N* → ∞. This implies that the effect of altered motility in dense regular graphs cannot be easily quantified in terms of a single parameter (the total number of edges). Then, motivated by tissue organization in multicellular organisms, in section Lattice graphs we consider one- and two-dimensional lattices. We show that in this setting, the difference in motility can be quantified and it has analogous effect to a difference in reproductive rate: increased motility results in mutant fixation with constant probability, whereas decreased motility causes the fixation probability to be exponentially small.

### 2.4. Related work

The question of computing fixation probabilities for various versions of Moran processes on graphs has been studied extensively. In principle, for any population structure the fixation probability can be computed numerically by solving a system of linear equations [62]. However, since the size of the system is generally exponential in the population size, this approach is practically feasible only for very small populations, or for very specific population structures [28,63,64]. For large population sizes, there exist efficient approximation algorithms either in the limit of weak selection [18,29,65,66] or when the underlying graph is undirected [15,67]. The two-graph setting was introduced recently by Melissourgos *et al.* [44], who considered it primarily from the computational perspective, and extended the above algorithmic results to a special case of mutants with substantial reproductive advantage (*r*_*A*_ ≫ *r*_*B*_) which perceive the population as a Complete graph (*G*_*A*_ = *K*_*N*_). They also considered certain game-theoretical perspective in which residents and mutants can choose their own graph structure, and they established bounds for certain specific pairs of graphs, such as the Complete graph invading the Star graph. By contrast, in this work we consider the two-graph setting from a biological perspective, and we present structural results concerning mutants with no reproductive advantage (*r*_*A*_ = *r*_*B*_) who, similarly to the residents, perceive the population structure either as an island model or as a low-dimensional lattice. On top of that, we answer a question stated in [44] related to the best-response dynamics in the space of all graphs. Namely, we show that while the Complete graph is locally optimal (see theorem A.1 in appendix A), it is not always the best response (figure 9).

## 3. Results

### 3.1. Small graphs

In this section, we consider population structures on *N* labelled nodes, for small values of *N*. In this regime, the fixation probability *ρ*(*G*^*A*^, *G*^*B*^) can be computed exactly, by numerically solving a system of 2^*N*^ linear equations following standard methods [62].

**Figure 3.**
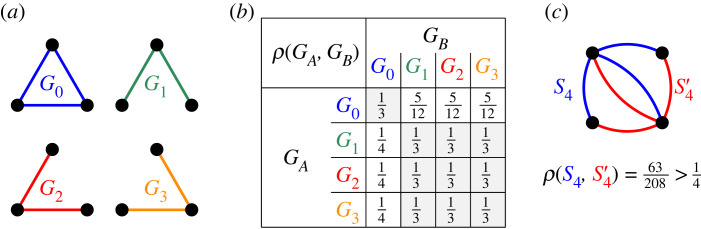
Small populations *N* = 3. (*a*) There are four connected graphs *G*^0^, …, *G*^3^ on *N* = 3 labelled nodes. (*b*) The fixation probabilities *ρ*(*G*^*A*^, *G*^*B*^) for all 4 · 4 = 16 combinations. (*c*) When *G*^*A*^ and *G*^*B*^ are isomorphic but not identical, the fixation probability is not necessarily equal to 1/*N*. For instance, we have $\rho (S_{4},S_{4}^{\prime })=63/208≐0.303$.

For *N* = 2, there is only one connected graph and, by symmetry, the fixation probability of a single type *A* individual is equal to 1/2. For *N* = 3, there are four undirected graphs: a single graph *G*^0^ with three edges (equivalently a Complete graph, or a Cycle), and three different graphs *G*^1^, *G*^2^, *G*^3^ with two edges each. The corresponding fixation probabilities are given in figure 3*b*. Note that *ρ*(*G*^*A*^, *G*^*B*^) = 1/*N* when *G*^*A*^ and *G*^*B*^ are identical, but in general *ρ*(*G*^*A*^, *G*^*B*^) could be both more than 1/*N* or less than 1/*N*, even when *G*^*A*^ and *G*^*B*^ are isomorphic (if they are not identical), see figure 3*c*.

**Figure 4.**
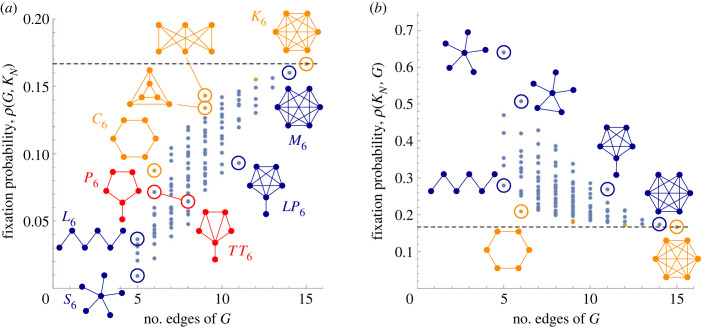
Small populations *N* = 6. The fixation probabilities (*a*) *ρ*(*G*) = *ρ*(*G*, *K*_*N*_) (residents on a complete graph) and (*b*) $\rho^{⋆}(G)=\rho (K_{N},G)$ (mutants on a complete graph) for all 112 graphs *G* on *N* = 6 nodes. Each dot corresponds to a graph *G*; the orange dots correspond to regular graphs. When *G* = *K*_*N*_, both *ρ*(*G*) and $\rho^{⋆}(G)$ are equal to 1/*N*. Other graphs *G*_6_ on six nodes satisfy *ρ*(*G*_6_) < 1/6 and $\rho^{⋆}(G_{6})>1/6$.

For general *N*, there are $2^{N^{2}-N}$ pairs of graphs on *N* labelled nodes. Already for *N* = 6 this is more than a billion pairs, hence in what follows we focus on the case when one of the graphs *G*^*A*^, *G*^*B*^ is a Complete graph, denoted *K*_*N*_. We use a short-hand notation *ρ*(*G*) = *ρ*(*G*, *K*_*N*_), for the fixation probability of a single mutant which perceives the population structure as a graph *G* and invades a population of residents which perceive the population structure as a Complete graph *K*_*N*_. Analogously, we denote by $\rho^{⋆}(G)=\rho (K_{N},G)$ the fixation probability of a single mutant living on a Complete graph *K*_*N*_ and invading a population of residents which live on *G*. Figure 4 shows *ρ*(*G*) and $\rho^{⋆}(G)$ for all undirected graphs on *N* = 6 nodes, based on the number of edges in *G*.

### 3.2. Maximal and minimal fixation probability

Among the graphs on six nodes, fixation probability *ρ*(*G*) is maximized when *G* is the Complete graph *K*_6_. Recall that *ρ*(*K*_*N*_) = 1/*N*, for any integer *N*. In relation to this, we prove that *ρ*(*K*_*N*_) is ‘locally maximal’: that is, we show that if one edge is removed from the Complete graph *K*_*N*_, then the resulting graph *M*_*N*_ satisfies *ρ*(*M*_*N*_) = (*N* − 2)/(*N* − 1)^2^ < 1/*N* = *ρ*(*K*_*N*_). Similarly, we prove that *K*_*N*_ is locally minimal with respect to $\rho^{⋆}(G)$: we show that $\rho^{⋆}(M_{N})=1/(N-1)>1/N$, see theorem A.1 in appendix A.

Note that, in contrast, for *N* = 6 the fixation probability *ρ*(*G*) is minimized for the Star graph *S*_6_. Here a *Star graph*, denoted *S*_*N*_, consists of a single node (called *centre*) connected to all other nodes (called *leaves*). It is known [44] that *ρ*(*S*_*N*_) ≤ 1/(*N* − 2)! and $\rho^{⋆}(S_{N})\to 1$ as *N* → ∞.

### 3.3. Relation to the number of edges

In general, fixation probability *ρ*(*G*) tends to be higher for graphs *G* with more edges. However, this is only a rule of thumb. For instance, the Lollipop graph *LP*_6_ has a relatively low fixation probability *ρ*(*LP*_6_), given its number of edges. Here a *Lollipop graph*, denoted *LP*_*N*_, consists of a Complete graph on *N* − 1 nodes and a single extra edge connecting the last node. Moreover, adding edges to a graph *G* to produce a graph *G*′ sometimes does not increase the fixation probability but rather decreases it: this is illustrated by the Pan graph *P*_6_ and the Treetop graph *TT*_6_ for which we have *ρ*(*P*_6_) > 0.071 and *ρ*(*TT*_6_) < 0.065. Here a *Pan graph*, denoted *P*_*N*_, consists of a cycle on *N* − 1 nodes and a single extra edge connecting the last node. In a *Treetop graph*, denoted *TT*_*N*_, the node with degree 3 is further connected to all other nodes. (We note that for the intermediate graph *I*_6_ obtained by adding only one edge to *P*_6_, the fixation probability *ρ*(*I*_6_) ≈ 0.070 is in between those two values.)

### 3.4. Regular graphs

Recall that a graph is *regular* if all its nodes have the same degree (that is, the same number of neighbours). We make two observations.

First, recall that when both the mutants and the residents perceive the population structure as the same regular graph *R*_*N*_, the isothermal theorem of [12] states that the mutant fixation probability is equal to (1 − 1/*r*)/(1 − 1/*r*^*N*^), where *r* is the mutant fitness advantage. That is, in terms of the fixation probability, all regular graphs are indistinguishable from each other. One could hope that the same holds for mutants which live on regular graphs and invade the residents on the Complete graph. That is, one could hope that for any two regular graphs *R*_*N*_, *R*′_*N*_ we have *ρ*(*R*_*N*_, *K*_*N*_) = *ρ*(*R*′_*N*_, *K*_*N*_). However, figure 4 shows that this generalization does not hold: for two different regular graphs *R*_*N*_ and *R*′_*N*_ (even with the same degree) the fixation probabilities *ρ*(*R*_*N*_, *K*_*N*_) and *ρ*(*R*_*N*_′, *K*_*N*_) are generally different, as witnessed by the two 3-regular graphs with *N* = 6 nodes and 9 edges.

Second, figure 4 shows that, given a fixed number of edges, the fixation probability *ρ*(*G*) is higher for regular (or almost regular) graphs as compared with non-regular graphs. For instance, among the connected graphs with six edges the fixation probability is maximized by the Cycle graph *C*_6_ which is regular. Similarly, among the graphs with five edges it is maximized by the Line graph *L*_6_ which is almost regular. Here a *Cycle graph*, denoted *C*_*N*_, is the connected graph where each node is connected to two neighbours, and a *Line graph*, denoted *L*_*N*_, is the Cycle graph with one edge missing. However, we prove that the Line graph *L*_*N*_ generally does *not* maximize the fixation probability among the connected graphs with *N* − 1 edges (so-called trees): in particular, direct computation for *N* = 8 shows that the graph *G*_8_ consisting of three paths of lengths 2, 2 and 3 meeting at a single node satisfies *ρ*(*G*_8_) > 0.0098 > 0.0095 > *ρ*(*L*_8_).

### 3.5. Dense regular graphs

As suggested by figure 4, regular graphs *G* have high fixation probability *ρ*(*G*), compared with other graphs with the same number of edges. Here we consider certain simple regular graphs that contain approximately half of the total possible number of edges. We show that for some such graphs, the fixation probability is comparable to that of a Complete graph, whereas for other graphs it is substantially smaller. Thus, the isothermal theorem [12] does not generalize to the setting with two different graphs.

**Figure 5.**
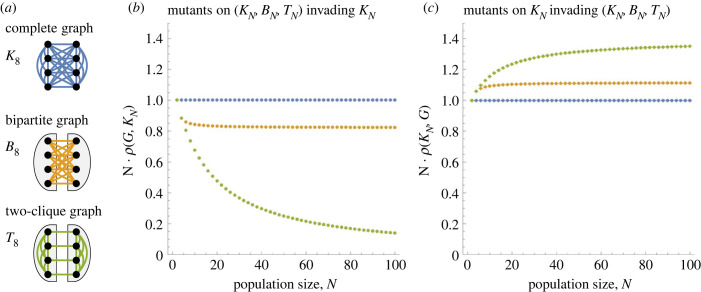
Dense regular graphs. (*a*) In a (complete) Bipartite graph *B*_*N*_ and a Two-clique graph *T*_*N*_, each node is connected to *N*/2 other nodes (here *N* is even). (*b*) When the mutant lives on *B*_*N*_, the fixation probability satisfies *ρ*(*B*_*N*_) ≈ 0.82 · 1/*N*. By contrast, when the mutant lives on *T*_*N*_, the fixation probability *ρ*(*T*_*N*_) tends to zero faster than 1/*N*. (*c*) When the residents live on *B*_*N*_ or *T*_*N*_, we have $\rho^{⋆}(B_{N})\approx 1.1\cdot 1/N$ and $\rho^{⋆}(T_{N})\approx 1.4\cdot 1/N$.

Given a population size *N* (with *N* even), let *B*_*N*_ = *K*_*N*/2,*N*/2_ be a (complete) *Bipartite graph* with equal parts *N*/2, *N*/2 and let *T*_*N*_ be a *Two-clique* graph obtained by adding *N*/2 matching edges to a union of two disjoint Complete graphs of size *N*/2 each, see figure 5*a*. Note that both *B*_*N*_ and *T*_*N*_ have precisely (1/4)*N*^2^ edges, which is roughly half of the edges of *K*_*N*_. (The graph *K*_*N*_ has (1/2)*N*(*N* − 1) ≈ (1/2)*N*^2^ edges.) Also, note that both *B*_*N*_ and *T*_*N*_ represent populations subdivided into two large islands: in the case of *B*_*N*_, the offspring always migrates to the opposite island, whereas in the case of *T*_*N*_, the offspring mostly stays in the same island and it migrates only rarely (namely with probability of the order of 1/*N*).

We prove that *ρ*(*B*_*N*_) > 0.58/*N* (see theorem B.1 in appendix B). Since *ρ*(*K*_*N*_) = 1/*N*, this implies that missing roughly half of the edges only reduces the fixation probability by a constant factor, independent of the population size *N*. In fact, numerical computation shows that *N* · *ρ*(*B*_*N*_) ≈ 0.82, whereas for the Two-clique graph we observe *N* · *ρ*(*T*_*N*_) → 0, see figure 5*b*.

The intuition for this distinction is as follows. On both graphs, the state of the system at any given time point is completely described by the frequencies *N*_*L*_ ∈ [0, *N*] and *N*_*R*_ ∈ [0, *N*] of mutants in the left and the right half. On *B*_*N*_, the two frequencies remain roughly equal throughout the process (*N*_*L*_ ≈ *N*_*R*_): indeed, once say *N*_*L*_ ≫ *N*_*R*_, more mutant offspring is produced on the left and they migrate to the right, thereby helping balance the numbers again. By contrast, on *T*_*N*_ the mutants migrate rarely, thus the lineage produced by the initial mutant remains trapped in one half for substantial amount of time. Throughout that time, the mutants are ‘blocking’ each other from spreading more than they would block each other if they were split evenly between the two halves: indeed, with all mutants in one half, the probability that a reproducing mutant replaces another mutant (thus not increasing the size of the mutant subpopulation) is twice as large, as compared with the situation where the mutants are evenly split. For small mutant subpopulations, this effect is non-negligible and it causes the fixation probability *ρ*(*B*_*N*_) to decay faster than inversely proportionally to *N*.

Regarding $\rho^{⋆}$, we observe $N\cdot \rho^{⋆}(B_{N})\approx 1.11$ and the data on $N\cdot \rho^{⋆}(T_{N})$ is unclear, see figure 5*c*. While we believe that $N\cdot \rho^{⋆}(T_{N})\approx 1.4$, it is conceivable that $N\cdot \rho^{⋆}(T_{N})$ tends to a larger constant or even grows unbounded. The intuition is that when mutants live on a Complete graph *K*_*N*_, the offspring is equally likely to migrate to any location. By randomness, the condition *N*_*L*_ ≈ *N*_*R*_ is thus maintained throughout most of the early stages of the process. Therefore, as with *ρ*(*B*_*N*_), both $\rho^{⋆}(B_{N})$ and $\rho^{⋆}(T_{N})$ are inversely proportional to *N*. To sum up, the graphs *B*_*N*_ and *T*_*N*_ show a considerably different behaviour in terms of *ρ* but a qualitatively comparable behaviour in terms of $\rho^{⋆}$.

### 3.6. Lattice graphs

Here we study sparse regular graphs, specifically lattice graphs. Lattices exist in any number of dimensions. We focus on one- and two-dimensional lattices, since those are biologically relevant and amenable to our computational techniques. For each dimension, we study the effect of increased or decreased connectivity (degree) of the lattice on the fixation probability of an invading mutant.

#### 3.6.1. One-dimensional lattices

**Figure 6.**
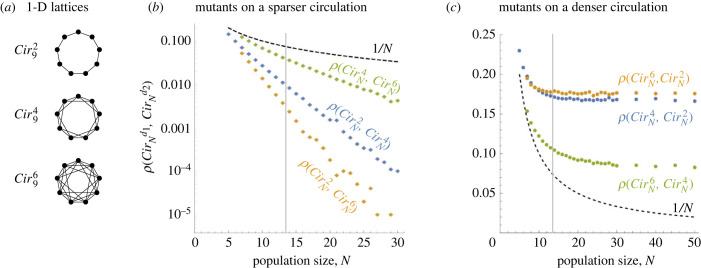
Overlaying one-dimensional lattices with different connectivities. (*a*) A circulation graph ${\mathrm{Cir}}_{N}^{d}$ is a one-dimensional lattice with periodic boundary and connectivity (degree) *d*. We consider *d* ∈ {2, 4, 6}. (*b*) When mutants live on a less connected graph (*d*_1_ < *d*_2_), their fixation probability decays to 0 at an exponential rate as *N* → ∞ (here *y*-axis is log-scale). (*c*) By contrast, when mutants live on a more densely connected graph (*d*_1_ > *d*_2_), their fixation probability tends to a constant. In both panels, the black dashed line shows the neutral baseline 1/*N*. The values for *N* ≤ 13 are computed by numerically solving a large system of linear equations. The values for *N* ≥ 14 are obtained by simulating the process 10^5^ times and reporting the proportion of the runs that terminated with the mutant fixating rather than going extinct.

In one dimension, we consider circulation graphs ${\mathrm{Cir}}_{N}^{d}$ (already studied in this context from a different point of view, see [44]). For a fixed even integer *d*, a **d*-Circulation* graph, denoted ${\mathrm{Cir}}_{N}^{d}$, consists of *N* nodes arranged in a cycle, where each node is connected to *d* other nodes, namely the next *d*/2 nodes and the previous *d*/2 nodes in the cyclic order, see figure 6*a*.

To shorten the notation, we denote by $\rho_{N}^{\mathrm{1D}}(d_{1},d_{2})=\rho ({\mathrm{Cir}}_{N}^{d_{1}},{\mathrm{Cir}}_{N}^{d_{2}})$ the fixation probability of a mutant living on a one-dimensional lattice ${\mathrm{Cir}}_{N}^{d_{1}}$ with degree *d*_1_ versus a population of residents living on a one-dimensional lattice ${\mathrm{Cir}}_{N}^{d_{2}}$ with degree *d*_2_. Note that when *d*_1_ = *d*_2_ = *d* then $\rho_{N}^{\mathrm{1D}}(d,d)=1/N$.

When the degrees *d*_1_, *d*_2_ of the mutant and resident graph differ, the fixation probability crucially depends on which of the two degrees is larger. When the mutant graph has a lower connectivity (*d*_1_ < *d*_2_) then $\rho_{N}^{\mathrm{1D}}(d_{1},d_{2})$ tends to 0 exponentially quickly as *N* → ∞, see figure 6*b*. By contrast, when the mutant graph has a higher connectivity (*d*_1_ > *d*_2_) then $\rho_{N}^{\mathrm{1D}}(d_{1},d_{2})$ tends to a positive constant *c* that depends on *d*_1_ and *d*_2_, see figure 6c. Specifically, for large *N* we observe that $\rho_{N}^{\mathrm{1D}}(4,2)\approx 0.16$, $\rho_{N}^{\mathrm{1D}}(6,2)\approx 0.17$ and $\rho_{N}^{\mathrm{1D}}(6,4)\approx 0.09$.

Those results are in agreement with bounds 0.11≤ $\rho_{N}^{\mathrm{1D}}(4,2)\leq 0.25$ that we prove analytically by a stochastic domination argument (see theorem C.1 in appendix C). The intuition behind the argument is that once the mutants form a contiguous block of a large size, the block is more likely to expand rather than to diminish at both interfaces. Indeed, the probability of gaining the boundary node is the same as losing the (other) boundary node but, on top of that, mutants could skip the boundary node, invade the interior of the resident territory and only after that gain the skipped node. This event has a non-negligible probability of happening, hence there is a positive bias favouring the spread of mutants. For a formal proof, see theorem C.1 in appendix C.

#### 3.6.2. Two-dimensional lattices

**Figure 7.**
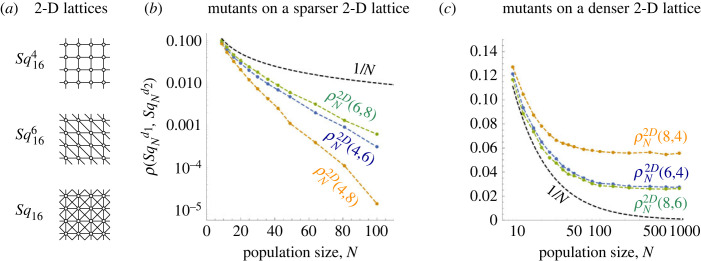
Overlaying two-dimensional lattices with different connectivities. (*a*) We consider two-dimensional lattices with degree 4 (Vonn Neumann neighbourhood), 6 (triangular grid) and 8 (Moore neighbourhood), and with dimensions *a* × *a* and *a* × (*a* + 1) for a=3,4,… ,30. (*b*,*c*) Similar to the one-dimensional case, the fixation probability decays to 0 exponentially quickly when *d*_1_ < *d*_2_, whereas it tends to a positive constant when *d*_1_ > *d*_2_. The black dashed line shows the baseline 1/*N*. The values are obtained by simulating the process (at least 10^5^ repetitions per data point).

In two dimensions, we consider graphs drawn on a square lattice with periodic boundary condition. For instance, by connecting each node to its four closest nodes (Von Neumann neighbourhood), we obtain a graph ${\mathrm{Sq}}_{N}^{4}$, see figure 7*a*. Similarly, by connecting to eight closest nodes (Moore neighbourhood) we obtain a graph ${\mathrm{Sq}}_{N}^{8}$. We also consider other graphs ${\mathrm{Sq}}_{N}^{d}$ with different connectivities *d* ∈ {6, 12, 20}. We again shorten the notation by denoting $\rho_{N}^{\mathrm{2D}}(d_{1},d_{2})=\rho ({\mathrm{Sq}}_{N}^{d_{1}},{\mathrm{Sq}}_{N}^{d_{2}})$.

The results are analogous to the case of one-dimensional lattices. When the mutants live on a less connected lattice, their fixation probability tends to 0 exponentially quickly. By contrast, when they live on a more densely connected lattice, their fixation probability tends to a constant as the population size *N* tends to infinity (figure 7).

#### 3.6.3. Effective fitness compared with complete graphs.

The behaviour of the fixation probability for pairs of low-dimensional lattices is reminiscent of the behaviour of the fixation probability *ρ*(*K*_*N*_; *r*) of a single mutant with relative reproductive rate *r* ≠ 1 in a well-mixed population of *N* − 1 other residents. In that setting, we have *ρ*(*K*_*N*_; *r*) = (1 − 1/*r*)/(1 − 1/*r*^*N*^). For any fixed *r* ≠ 1, this formula exhibits one of two possible behaviours in the limit *N* → ∞. When *r* < 1 then *ρ*(*K*_*N*_; *r*) decays approximately as 1/*r*^*N*^. By contrast, when *r* > 1 then it tends to a positive constant 1 − 1/*r*. (When *r* = 1 we have *ρ*(*K*_*N*_; *r*) = 1/*N* by symmetry.) This is a similar dichotomy to the one we observed in the case when mutants and residents perceive the population structure as two lattices with different connectivities.

In other words, enabling a neutral mutant to live on a more densely connected lattice has a comparable effect on its fixation probability as giving it a certain relative reproductive advantage. Formally, given a population size *N* and two lattices *L*_*N*_, *L*′_*N*_ we define the *effective fitness*, denoted *r*(*L*_*N*_, *L*′_*N*_), as the unique number *r* such that

$$\rho (L_{N},L_{N}^{\prime })=\rho (K_{N};r).$$

In other words, the effective fitness is such a number *r*(*L*_*N*_, *L*′_*N*_), that a neutral mutant on a lattice *L*_*N*_ invading a lattice *L*′_*N*_ has the same fixation probability as a mutant with relative reproductive advantage *r*(*L*_*N*_, *L*′_*N*_) in a well-mixed population.

**Figure 8.**
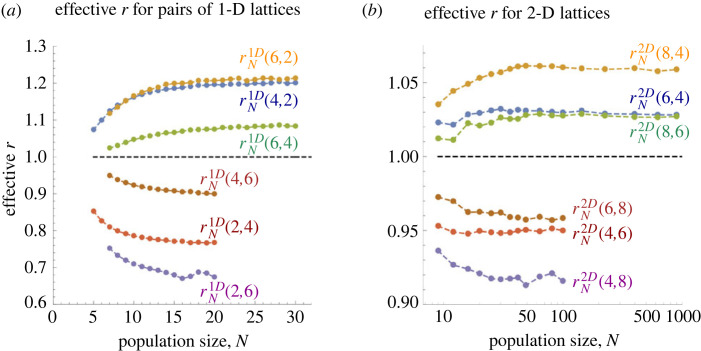
Effective fitness compared with complete graphs. Given the connectivities *d*, *d*′ of the mutant and resident lattice, we compute the effective fitness that would result in the same fixation probability, had both types lived on a Complete graph. (*a*) One-dimensional lattices ${\mathrm{Cir}}_{N}^{d}$ with *d* ∈ {2, 4, 6}. (*b*) Two-dimensional lattices ${\mathrm{Sq}}_{N}^{d}$ for *d* ∈ {4, 6, 8}. We have $r_{N}^{\mathrm{2D}}(8,4)\approx 1.06$, $r_{N}^{\mathrm{2D}}(6,4)\approx r_{N}^{\mathrm{2D}}(8,6)\approx 1.03$, and $r_{N}^{\mathrm{2D}}(6,8)\approx 0.96$, $r_{N}^{\mathrm{2D}}(4,6)\approx 0.95$, $r_{N}^{\mathrm{2D}}(4,8)\approx 0.92$. In both panels, the black dashed line shows the neutral baseline *r* = 1.

For pairs of low-dimensional lattices with different connectivities *d*, *d*′, the effective fitness can be computed from the data presented above, see figure 8. We observe that while the effective fitness depends on the connectivities *d*, *d*′ of the two lattices and on their dimensionality, it is mostly independent of the population size *N*.

## 4. Discussion

In this work, we studied the effect of mutations that, rather than altering the reproductive rate of the affected individual, alter how the individual experiences the population structure. To that end, we considered a powerful framework based on the classical Moran birth–death process on graphs, in which the two types of individuals (the novel mutant and the existing residents) perceive the population structure through different graphs. Past evolutionary graph models have discussed inclusion of motility potential as a secondary parameter, besides reproductive rate or fitness [54,56]. Our approach generalizes this to the case where the whole migration matrix is genotype dependent, and thus population structure is determined by two graph structures, each for one competing genotype.

As the key quantity, we studied the probability *ρ*(*G*^*A*^, *G*^*B*^) that a single neutral mutant which perceives the population structure as a graph *G*^*A*^ successfully invades the population of residents which perceive the population structure as a graph *G*^*B*^. For small population sizes, we computed the pairwise fixation probabilities numerically, and we observed that *ρ*(*G*^*A*^, *G*^*B*^) tends to be higher when *G*^*A*^ is regular and when it contains many edges (that is, the mutant is more motile). We note that the latter aspect contrasts with other models of motility, where an increased dispersal potential of the mutant generally diminishes the fixation probability [55,57,58].

Next, motivated by island models, we considered two regular graphs with the same total number of edges, and we showed that the corresponding fixation probabilities are asymptotically different. In particular, as the population size *N* increases, the fixation probabilities decay at different rates. Thus, the classic isothermal theorem of [12] does not translate over to the setting in which population structures are type-specific.

Finally, we studied the biologically relevant cases of one- and two-dimensional lattices and we showed that the dispersal radius has similar effect on the fixation probability as the reproductive rate. Recall that in large unstructured populations, a beneficial mutation fixates with constant probability, whereas the fixation probability of a deleterious mutation is exponentially small. Likewise, neutral mutants on lattices with larger dispersal radius have a constant chance of successfully fixating, whereas having lower dispersal radius leads to fixation of the mutant only with exponentially small probability. Thus, in terms of the fixation probability of the mutant, perceiving the population through a more densely connected lattice is effectively equivalent to having an increased reproductive rate.

We view the presented model as a generalization of selection dynamics that is not only needed, but also necessary. For example, for the multicellular structures organized in tissues, the extracellular matrix is produced by a complex mechanism which involves the epithelial cells themselves. To highlight the effect of structural differences between two competing types, we primarily considered the case that mutants might not have different reproductive rate, but have a somewhat altered extracellular matrix. An important example is evolution of carcinoma in epithelial tissues. Malignant tumour cells cannot form the same ligands needed for ECM in a particular tissue. Instead, the organization of tumour cells is often reminiscent of random aggregation of cells or an unstructured mesh. This difference in population structure of normal cells and tumour cells can confer significant advantage, or disadvantage, for the tumour cells invading the tissue. The tissue mechanics has been studied in physical models in the past. Recently, selection dynamics has been studied using biomechanical models of tissue formation [68].

In this work, we consider the birth–death dynamics where an individual is selected for reproduction and the offspring replaces one neighbour. Alternatively, one could consider a death–birth version of the model, where an individual is chosen to die first and then one neighbouring cell replaces the void created with an offspring [12,18]. Notice that even in the neutral limit of *r*_*A*_ = *r*_*B*_, the two update rules would yield different results [69–72]. This has to do with the fact that effective fitness of each genotype depends not only on the reproduction rate, but also on the number of neighbours each genotype sees in its neighbourhood. On average, if a type *A* perceives more neighbours as a given location it will have higher chance to replace a neighbouring type *B*. We leave the study of death–birth process in this context for future work.

Moving on to more complex (though perhaps less realistic) population structures, many natural questions arise. We conclude by commenting on three of them. Recall that for any graph *G*_*N*_ on *N* nodes we have *ρ*(*G*_*N*_, *G*_*N*_) = 1/*N* [61].

First, figure 4 suggests that $\rho (G_{N}^{A},K_{N})<1/N$ for all mutant graphs $G_{N}^{A}\neq K_{N}$. While we can prove that *ρ*(*M*_*N*_, *K*_*N*_) < 1/*N* for a graph *M*_*N*_ that misses a single edge (see theorem A.1 in appendix A), the general claim is left as an open problem. Similarly, we do not know whether $\rho (K_{N},G_{N}^{B})>1/N$ holds for all resident graphs $G_{N}^{B}\neq K_{N}$ (we do know that it holds for $G_{N}^{B}=M_{N}$).

**Figure 9.**
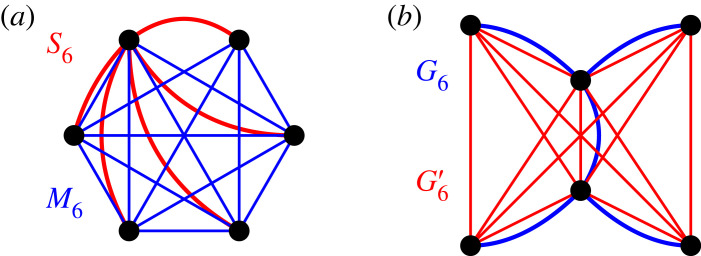
Best-response graphs. The Complete graph is sometimes not the best response when optimizing the fixation probability *ρ*. (*a*) The resident population living on the Star graph *S*_6_ (red) is easier to invade by mutants living on *M*_6_ (blue) than by mutants living on the Complete graph *K*_6_. (*b*) The mutants living on a graph *G*_6_ (blue) have a harder time invading the graph *G*′_6_ than they have invading the Complete graph *K*_6_.

Second, following the game theory perspective, Melissourgos *et al.* [44] asked what is the best mutant response to a given resident graph. That is, given a resident graph $G_{N}^{B}$ on *N* nodes, which mutant graph $G_{N}^{A}$ on *N* nodes maximizes the fixation probability $\rho (G_{N}^{A},G_{N}^{B})$? Our results for small graphs show that although the Complete graph *K*_*N*_ is frequently the best mutant response, it is not always the case, see figure 9. In particular, when the residents live on a Star graph *S*_6_, the population is easier to invade through a graph *M*_6_ that misses a single edge, rather than through the Complete graph *K*_6_—direct computation gives *ρ*(*M*_6_, *S*_6_) > 0.643 > 0.641 > *ρ*(*K*_6_, *S*_6_). We note that the difference is minor—both mutant graphs *M*_6_ and *K*_6_ provide a fixation probability well over the neutral threshold value 1/6 ≈ 0.167.

For the complementary question of what is the best resident response $G_{N}^{B}$ to a given mutant graph $G_{N}^{A}$, the situation is analogous: while the Complete graph is generally hard to invade, it is sometimes not the hardest one. As an example (see figure 9*b*), when mutants live on a graph *G*_6_ then for the graph *G*′_6_ we have *ρ*(*G*_6_, *G*′_6_) < 0.025 < 0.026 < *ρ*(*G*_6_, *K*_6_).

Third, consider the case when mutants and residents live on different graphs $G_{N}^{A}\neq G_{N}^{B}$ with comparable edge densities. In this case, we expect that fixation probabilities $\rho (G_{N}^{A},G_{N}^{B})$ and $\rho (G_{N}^{B},G_{N}^{A})$ typically drop below 1/*N*. The intuition is that the mutant subpopulation tends to form clusters in $G_{N}^{A}$ but not necessarily in $G_{N}^{B}$. As a consequence, mutants block each other from spawning onto residents but they do not guard each other from being replaced by residents. However, sometimes the opposite inequality $\rho (G_{N}^{A},G_{N}^{B})>1/N$ holds and in general the strength of the effect appears difficult to quantify. In particular, we do not know the answer to the following question: do there exist two regular graphs such that both *ρ*(*G*_*N*_, *G*′_*N*_) > 1/*N* and *ρ*(*G*′_*N*_, *G*_*N*_) > 1/*N*? We note that if the word ‘regular’ is dropped, the answer is ‘no’, as witnessed by the two Star graphs depicted in figure 3.

In conclusion, we have introduced a novel model of stochastic selection dynamics in structured populations, where motility potential for each type is reflected by how that type sees the neighbourhood. This has potential importance for modelling dynamics of aggressive solid tumours in epithelial tissues. The generalized theoretical framework corresponds to a migration matrix or evolutionary graph structure specific to each genotype. Our main observation is that, as a rule of thumb, if a type perceives a higher number of neighbours then it has a selection advantage. We also highlight several counter-examples, especially in small population sizes. Another important observation is that if the two graphs are regular and have the same degree connectivity, the fixation probability is not necessarily 1/*N*, but its value depends on how the two graphs are structured and overlaid. This might be somewhat surprising, from the perspective of the isothermal theorem. For a more applied case of large lattices, we report the equivalent fitness advantage that a mutant type gains by perceiving the population structure through a more densely connected lattice. Our current model and results can be the basis of a more biophysically inspired model of cancer progression where differences between normal and tumour extra-cellular matrix are included.

## Figures and Tables

**Figure 10.**
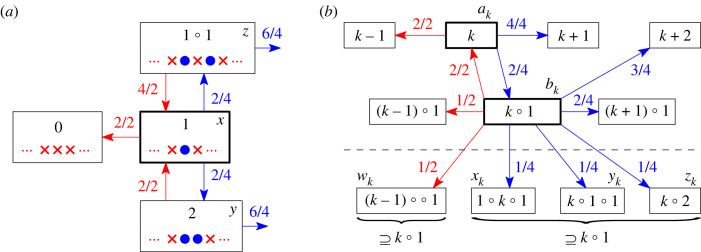
Proof of theorem C.1. (*a*) Upper bound. Boxes represent configurations. Mutants are shown as blue discs, residents as red crosses. Blue (red) transitions correspond to mutants (residents) reproducing. A fraction *a*/*b* denotes that there exist *a* relevant edges, each pointing from a vertex with degree *b*. With a constant probability, the random evolutionary trajectory leads to mutant extinction without ever reaching a configuration with three or more mutants. (*b*) Lower bound. We consider a process *M*^↓^ where, any time a configuration below the dashed line is reached, we remove a mutant to instead reach one of the configurations above the dashed line. We then compute the fixation probability in *M*^↓^, which is a lower bound.

**Figure 11.**
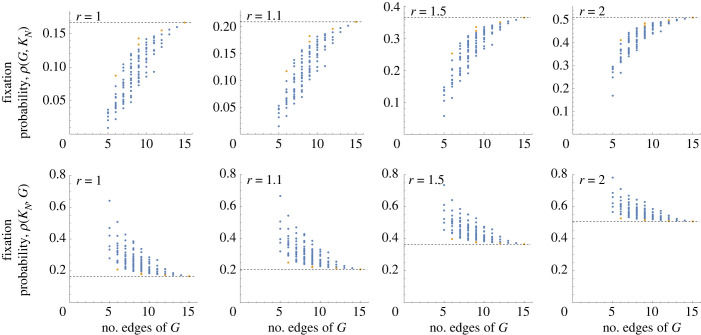
Small populations *N* = 6 with *r* ≥ 1. The fixation probabilities *ρ*(*G*) = *ρ*(*G*, *K*_*N*_) (top row) and $\rho^{⋆}(G)=\rho (K_{N},G)$ (bottom row) for all 112 graphs *G* on *N* = 6 nodes, with *r* ∈ {1, 1.1, 1.5, 2} (columns). As in figure 4, each dot corresponds to a graph *G* and the orange dots correspond to regular graphs. The shape of the point cloud is roughly preserved even as *r* increases.

## Data Availability

The datasets generated during and/or analysed during the current study are available as part of the electronic supplementary material: http://doi.org/10.6084/m9.figshare.16910170 [73].

## Appendix A. The Complete graph is locally optimal

Here we show that when it comes to *ρ*(*G*_*N*_) and $\rho^{⋆}(G_{N})$, the Complete graph is locally optimal. That is, we show that the graph *M*_*N*_ obtained from *K*_*N*_ by removing a single edge satisfies *ρ*(*M*_*N*_) = *ρ*(*M*_*N*_, *K*_*N*_) < 1/*N* and $\rho^{⋆}(M_{N})=\rho (K_{N},M_{N})>1/N$.

Theorem A.1. *Fix* *N* ≥ 2 *and let* *M*_*N*_ *be a graph obtained from the Complete graph* *K*_*N*_ *by removing a single edge. Then*

$$\rho (M_{N},K_{N})=\frac{N-2}{{\left(N-1\right)}^{2}}<\frac{1}{N}$$

*and*

$$\rho (K_{N},M_{N})=\frac{1}{N-1}>\frac{1}{N}.$$

Proof. Fix *N* ≥ 2. First we focus on *ρ*(*M*_*N*_) = *ρ*(*M*_*N*_, *K*_*N*_). Denote by φ(*a*, *b*) the fixation probability starting from a configuration with *a* mutants among the *N* − 2 fully connected vertices and *b* mutants among the other two vertices that miss one edge. The values {φ(*a*, *b*)| 0 ≤ *a* ≤ *N* − 2, 0 ≤ *b* ≤ 2} are the unique solution to the following system of linear equations:

— φ(0, 0) = 0;
— φ(*N* − 2, 2) = 1; and
— $\varphi (a,b)=\sum_{\left(a^{\prime },b^{\prime }\right)}p_{\left(a,b)\to (a^{\prime },b^{\prime }\right)}\cdot \varphi (a^{\prime },b^{\prime })$, where *p*_(*a*,*b*)→(*a*′,*b*′)_ is the probability that, in a single step of the Moran process, we transition to a configuration with *a*′ mutants among the *N* − 2 fully connected vertices, and *b* mutants among the other two vertices.

We claim that

$$\varphi (a,b)=\frac{\left(a+b)(n-2)+\frac{1}{2}b(b-1\right)}{{\left(n-1\right)}^{2}}\;$$

is a solution to this system. Clearly, the formula satisfies φ(0, 0) = 0 and φ(*n* − 2, 2) = 1. Since the equation in the last item in the list above can be rewritten as

$$\sum_{\left(a^{\prime },b^{\prime }\right)}p_{\left(a,b)\to (a^{\prime },b^{\prime }\right)}\cdot (\varphi (a^{\prime },b^{\prime })-\varphi (a,b))=0,$$

it suffices to check that given an arbitrary configuration (that is, any 0 ≤ *a* ≤ *n* − 2 and 0 ≤ *b* ≤ 2), the expected value of the formula after a single transition does not change. The transition probabilities are as follows:

(i) $p_{b+}\equiv \mathrm{Pr}[(a,b)\to (a,b+1)]=(a/n)\cdot (2-b)/(n-1)$,
(ii) $p_{a+}\equiv \mathrm{Pr}[(a,b)\to (a+1,b)]=(a/n)\cdot (n-2-a)/(n-1)+(b/n)\cdot (n-2-a)/(n-2)$,
(iii) $p_{b-}\equiv \mathrm{Pr}[(a,b)\to (a,b-1)]=(n-a-b)/n\cdot b/(n-1)$,
(iv) $p_{a-}\equiv \mathrm{Pr}[(a,b)\to (a-1,b)]=(n-a-b)/n\cdot a/(n-1)$.

Ignoring the shared denominator (*n* − 1)^2^, the expected change in the value produced by the formula in the respective cases is as follows:

(i) Δφ_*b*+_ = +*n* − 2 + *b*,
(ii) Δφ_*a*+_ = +*n* − 2,
(iii) Δφ_*b*−_ = −(*n* − 2 + *b* − 1),
(iv) Δφ_*a*+_ = −(*n* − 2).

Denoting *I* = {*b*+, *a*+, *b*−, *a*−} we compute

$$\begin{array}{rl} \Delta \varphi & \equiv \sum_{i\in I}p_{i}\cdot \Delta \varphi_{i}\\ & =\frac{1}{n(n-1)}\cdot [a(2-b)(n-2+b)+a(n-2-a)(n-2)\\ & \;+b(n-2-a)(n-1)-(n-a-b)b(n-2+b-1)\\ & \;-(n-a-b)a(n-2)]. \end{array}$$

Grouping the terms with *n* raised to the same power, the terms in the square brackets on the right-hand side can be rearranged as *n*^2^ · *X* + *n* · *Y* + *Z*, where *X* = *a* + *b* − *b* − *a* = 0, next

$$\begin{array}{rl} Y & =(2a-ab)+(-2a-a^{2}-2a)+(-2b-ab-b)\\ & \;-(-ab-b^{2}-2b+b^{2}-b)-(-2a-a^{2}-ab)=0, \end{array}$$

and finally

$$\begin{array}{rl} Z & =(-ab^{2}+4ab-4a)+2a^{2}+(ab+2b)\\ & \;+(b^{3}+ab^{2}-3b^{2}-3ab)-(2a^{2}+2ab)\\ & =b^{3}-3b^{2}+2b=b(b-1)(b-2). \end{array}$$

Since *Z* = 0 for any admissible integer *b* (recall 0 ≤ *b* ≤ 2), we are done. Regarding $\rho^{⋆}(M_{N})$, a completely analogous proof establishes a formula

$$\varphi (a,b)=\frac{(a+b)(n-2)+b(3-b)/2}{{\left(n-1\right)}^{2}},$$

which implies the desired

$$\rho^{⋆}(M_{N})=\frac{1\cdot (n-2)+\frac{1\cdot 2}{2}}{{\left(n-1\right)}^{2}}=\frac{1}{n-1}.\;\text{■}$$

## Appendix B. Bipartite graph *B*_*N*_ is within a constant factor of *K*_*N*_

Recall that for *N* even we denote by *B*_*N*_ the (complete) Bipartite graph with parts of sizes *N*/2 and *N*/2. We prove that for *N* large, both *ρ*(*B*_*N*_) and $\rho^{⋆}(B_{N})$ are within a constant factor of 1/*N*. Recall that numerical experiments in the main text suggest that *ρ*(*B*_*N*_) ≈ 0.82/*N* and $\rho^{⋆}(B_{N})\approx 1.1/N$, as *N* → ∞.

Theorem B.1. *Fix* *N* ≥ 2 *and let* *B*_*N*_ *the* (*complete*) *Bipartite graph with parts of sizes* *N*/2 *and* *N*/2. *Then*

$$\rho (B_{N})>\frac{1}{e-1}\cdot \frac{1}{N}\;\mathrm{and}\;\rho^{⋆}(B_{N})<\frac{e}{e-1}\cdot \frac{1}{N},$$

*where* e≐2.718… *is Euler’s number*.

Proof. First we bound *ρ*(*B*_*N*_). Consider a time point at which there are *n* mutants in total: *k* of them in one part and *n* − *k* in the other part. The probability *p*^+^(*k*, *n* − *k*) that in the next time step we gain a mutant equals

$$\begin{array}{rl} \;p^{+}(k,n-k) & =\frac{k}{N}\cdot \frac{N/2-(n-k)}{N/2}+\frac{n-k}{N}\cdot \frac{N/2-k}{N/2}\\ & =\frac{k(N-2n+2k)+(n-k)(N-2k)}{N^{2}}\\ & =\frac{n\cdot N-4k(n-k)}{N^{2}}. \end{array}$$

Since 4*k*(*n* − *k*) ≤ *n*^2^ for any k=0,…,n (and with equality only for *k* = *n*/2), we can bound

$$p^{+}(k,n-k)\geq \frac{n\cdot N-n^{2}}{N^{2}}=\frac{n(N-n)}{N^{2}}.$$

On the other hand, the probability *p*^−^(*k*, *n* − *k*) that in the next time step we lose a mutant equals

$$p^{-}(k,n-k)=\frac{N-n}{N}\cdot \frac{n}{N-1}$$

and thus

$$\frac{\;p^{-}(k,n-k)}{p^{+}(k,n-k)}\leq \frac{N}{N-1}\equiv t.$$

Since this expression is independent of *k* and *n*, plugging it in the standard formula for the absorption probability of a one-dimensional Markov chain we get

$$\rho (B_{N})\geq \frac{1}{1+\sum_{i=i}^{N-1}t^{i}}=\frac{t-1}{t^{N}-1}.$$

Since *t* − 1 = 1/(*N* − 1) > 1/*N* and

$$t^{N}={\left(\frac{N}{N-1}\right)}^{N}={\left(1+\frac{1}{N-1}\right)}^{N}\geq e,$$

where e≐2.718 is Euler’s number, we thus obtain

$$\rho (B_{N})>\frac{1}{e-1}\cdot \frac{1}{N}>\frac{0.58}{N}.$$

Regarding $\rho^{⋆}(B_{N})$, a completely analogous proof yields

$$p^{+}(k,n-k)=\frac{n}{N}\cdot \frac{N-n}{N-1}$$

and

$$p^{-}(k,n-k)=\frac{n\cdot N-4k(n-k)}{N^{2}}\geq \frac{n(N-n)}{N^{2}}.$$

Therefore,

$$\frac{\;p^{-}(k,n-k)}{p^{+}(k,n-k)}\geq \frac{N-1}{N}\equiv t.$$

Finally, since *t*^*N*^ = (1 − 1/*N*)^*N*^ ≤ 1/*e*, we get

$$\rho^{⋆}(B_{N})\leq \frac{t-1}{t^{N}-1}\leq \frac{1/N}{1-1/e}=\frac{e}{e-1}\cdot \frac{1}{N}.\text{■}$$

## Appendix C. One-dimensional lattices

Recall that for a fixed even integer *d* we denote by ${\mathrm{Cir}}_{N}^{d}$ the graph whose *N* vertices, labelled 1, …, *N*, are arranged along a circle, and each vertex is connected with *d*/2 closest vertices clockwise and *d*/2 closest vertices counter-clockwise. Also, recall that given two connectivities *d*_1_, *d*_2_ we shorthand

$$\rho_{N}^{\mathrm{1D}}(d_{1},d_{2})=\rho ({\mathrm{Cir}}_{N}^{d_{1}},{\mathrm{Cir}}_{N}^{d_{2}}).$$

Figure 6 suggests that when *d*_1_ > *d*_2_ then the expression $\rho_{N}^{\mathrm{1D}}(d_{1},d_{2})$ remains bounded away from 0 as *N* → ∞, Below we prove this in the special case (*d*_1_, *d*_2_) = (4, 2), that is, when the mutants can place their offspring on the next two vertices and on the preceding two vertices, whereas the residents can place their offspring only on the next one vertex and on the preceding one vertex.

Theorem C.1.
*We have*

$$0.138<\lim_{N\to \infty }\rho_{N}^{\mathrm{1D}}(4,2)<0.34.$$

Proof. By a *configuration*, we mean a subset of vertices occupied by mutants. We denote the possible configurations as a sequence of numbers (corresponding to blocks of consecutive mutants) and symbols ‘°’ (corresponding to individual residents). That is, for instance the notation *k* ° 1 denotes a configuration with *k* consecutive mutants, then one resident, then one more mutant (and residents before and after). *Proof of the upper bound.* This is straightforward. We say that a step of the Moran process is *active* if it changes the configuration. Given a single mutant, there are two possible active steps leading to immediate extinction (each occurs with probability equal to 1/*N* · 1/2). On the other hand, there are four possible active steps where mutants reproduce (each occurs with probability equal to 1/*N* · 1/4). Thus, in total, with probability (2/2)/(2/2 + 4/4) = 1/2 the first active step results in the mutant extinction, hence $\rho_{N}^{\mathrm{1D}}(4,2)\leq 1/2$. Accounting for trajectories that never reach a configuration with more than *m* mutants, we can push this upper bound lower: for instance, taking *m* = 2, denote by *x*, *y*, *z* the extinction probabilities from configurations 1, 2, 1 ° 1, respectively. Suppose that *N* ≥ 5 and we are currently at configuration 1. With probability (1/*N*) · 2/2, one neighbour of the single mutant is selected for reproduction and the offspring replaces the mutant, resulting in mutant extinction. With probability (1/*N*) · 2/4 the mutant reproduces onto a neighbour and we reach a configuration 2. Similarly, with probability (1/*N*) · 2/4 the mutant offspring ‘skips’ the neighbour, resulting in configuration 1 ° 1. Otherwise, we remain at configuration 1. Thus, conditioning on the first active step (figure 10*a*) we obtain

$$x=\frac{2/2\cdot 1+2/4\cdot y+2/4\cdot z}{\left(2/2)+(2/4)+(2/4\right)}=(1/2)+(1/4)y+(1/4)z.$$

Likewise, we get

$$y\geq \frac{6/4\cdot 0+2/2\cdot x}{\left(6/4)+(2/2\right)}=(2/5)x\;\mathrm{and}\;z\geq \frac{6/4\cdot 0+4/2\cdot x}{\left(6/4)+(4/2\right)}=(4/7)x,$$

hence *x* ≥ (1/2) + 1/4 · (2/5)*x* + 1/4 · (4/7)*x*. This rewrites as *x* ≥ 35/53, hence $\rho_{N}^{\mathrm{1D}}(4,2)\leq 18/53<0.34$ for any *N* ≥ 5. *Proof of the lower bound.* For *k* ≥ 1 denote by *a*_*k*_ the fixation probability from configuration *k*. Similarly, for *k* ≥ 1 denote by *b*_*k*_ the fixation probability from the configuration *k* ° 1. Then *a*_0_ = 0 and

$$a_{1}=\frac{(2/2)a_{0}+(2/4)a_{2}+(2/4)b_{1}}{\left(2/2)+(2/4)+(2/4\right)}=(1/4)(a_{2}+b_{1}).$$

For *k* ≥ 2 we have (figure 10*b*)

$$a_{k}=\frac{(2/2)a_{k-1}+(2/4)b_{k}+(4/4)a_{k+1}}{\left(2/2)+(2/4)+(4/4\right)}=(1/5)(2a_{k-1}+b_{k}+2a_{k+1}).$$

For analogous expressions of *b*_*k*_, we would need to introduce new variables *m*_*k*_, *x*_*k*_, *y*_*k*_, *z*_*k*_ to describe fixation probabilities from configurations listed below the dashed line. Instead of computing the fixation probability exactly, we bound it by considering a different processes *M*^↓^, where mutants have lower fixation probability than in the original process *M*. We define *M*^↓^ as follows: the two processes coincide, except that when *M* reaches any configuration below the dashed line, we remove several mutants so as to obtain a configuration *a*_*i*_ or *b*_*i*_ (for some *i*), see figure 10*b*. For instance, when the current configuration is *k* ° 1, the resident from the gap is spawning an offspring, and the offspring moves one place left resulting in a configuration (*k* − 1) °° 1, we additionally remove the single separated mutant, reaching the configuration *k* − 1, where the mutants have fixation probability *a*_*k*−1_. Since for any two configurations *C* ⊆ *C*′ we have *ρ*(*C*) ≤ *ρ*(*C*′), the fixation probability in *M*^↓^ is indeed decreased, as compared with *M*. (We note that by considering a process *M*^↑^ where we occasionally add mutants so as to only visit configurations *a*_*i*_, *b*_*i*_, one can obtain a stronger upper bound than the one presented above.) In *M*^↓^ we thus obtain

$$\begin{array}{rl} b_{k} & =\frac{(2/2)a_{k}+(1/2)b_{k-1}+(1/2)a_{k-1}+(3/4)b_{k}+(2/4)b_{k+1}+(3/4)a_{k+2}}{\left(2/2)+(1/2)+(1/2)+(3/4)+(2/4)+(3/4\right)}\\ & =\frac{1}{16}(2a_{k-1}+4a_{k}+3a_{k+2}+2b_{k-1}+3b_{k}+2b_{k+1}). \end{array}$$

It remains to compute the fixation probability in *M*^↓^, in the limit *N* → ∞. We do this by a standard argument. Consider a ‘potential function’ φ which assigns a positive real number to each configuration, defined by φ(*k*) = *α*^*k*^, φ(*k* ° 1) = *c* · *α*^*k*^, where *α*, *c* > 0 are positive real numbers (to be defined later). By solving a system of two equations

$$5=\frac{2}{\alpha }+c+2\alpha$$

and

$$16c=\frac{2}{\alpha }+4+3\alpha^{2}+\frac{2c}{\alpha }+3c+2c\alpha$$

we find values α≐0.860, c≐0.954 (the roots of certain degree-3 polynomials) for which the (expected) potential does not change in one step of the process (that is, the function φ is a martingale), except when at configuration 1. Now for any initial configuration *x* ≠ 1, run the process starting from *x* until it reaches either a configuration 1 or the configuration *N*, and let *p*_*x*_ be the probability that the former happens. Then

$$\begin{array}{rl} \varphi (x) & =E[\varphi (x)|\text{when\textbackslash{} the\textbackslash{} process\textbackslash{} ends}]\\ & =p_{x}\cdot \varphi (1)+(1-p_{x})\cdot \varphi (N), \end{array}$$

which rewrites as

$$p_{x}=\frac{\varphi (x)-\varphi (N)}{\varphi (1)-\varphi (N)}.$$

Since *α* < 1 we have lim _*N*→∞_φ(*N*) = 0, thus for *x* ∈ {2, 1 ° 1} we can write

$$\lim_{N\to \infty }p_{2}=\frac{\alpha^{2}}{\alpha }=\alpha \;\text{and}\;\lim_{N\to \infty }p_{1\circ 1}=\frac{c\cdot \alpha }{\alpha }=c.$$

Now using the expressions

$$a_{2}=p_{2}\cdot a_{1}+(1-p_{2})\cdot 1\;\mathrm{and}\;b_{2}=p_{1\circ 1}\cdot a_{1}+(1-p_{1\circ 1})\cdot 1$$

and plugging them into $a_{1}=\frac{1}{4}(a_{2}+b_{1})$ we finally obtain

$$a_{1}=\frac{1}{4}((2-p_{2}-p_{1\circ 1})+(p_{2}+p_{1\circ 1})\cdot a_{1}),$$

hence

$$\lim_{N\to \infty }a_{1}=\lim_{N\to \infty }\frac{2-p_{2}-p_{1\circ 1}}{4-p_{2}-p_{1\circ 1}}=\frac{2-\alpha -c}{4-\alpha -c}>0.138.\text{■}$$

## Appendix D. Edge-weighted graphs

Throughout the main text, we consider graphs in which each edge has unit weight. Moran process can be extended to graphs with weighted edges. Then, during each reproduction step, the offspring migrates along an adjacent edge chosen not uniformly at random, but rather with probability proportional to the corresponding edge weight. Since edge weights can be any non-negative real numbers, the space of all edge-weighted graphs is immense. To illustrate the difficulties when considering edge-weighted graphs, here we consider a special case, where *N* = 3, the residents live on a complete (unweighted) graph *K*_3_, and the mutants live on an edge-weighted graph $G_{3}^{x,y,z}$ with edge weights *x*, *y*, *z*. By solving the resulting system of linear equations symbolically, one can obtain the following unwieldy formula:

$$\rho (G_{3},K_{3})=\frac{4\sum_{6}x^{4}y^{2}+12\sum_{3}x^{4}yz+52\sum_{3}x^{3}y^{3}+193\sum_{6}x^{3}y^{2}z+426\sum_{1}x^{2}y^{2}z^{2}}{42\sum_{6}x^{4}y^{2}+96\sum_{3}x^{4}yz+156\sum_{3}x^{3}y^{3}+549\sum_{6}x^{3}y^{2}z+1098\sum_{1}x^{2}y^{2}z^{2}},$$

where each symbol $\sum_{i}$ denotes the sum of all *i* distinct terms obtained by permuting the exponents in the variables *x*, *y*, *z*.

To perform a sanity check, we consider a special case $G_{3}^{x,x,1}$, where the graph has two edges of equal weight *x* = *y* ∈ [0, 1] and one edge of unit weight. In that case, the formula simplifies into

$$\rho (G_{3}^{x,x,1},K_{3})=\frac{1+23x+6x^{2}}{9+57+24x^{2}}.$$

When *x* = 1, the graph $G_{3}^{x,x,1}$ is itself the complete unweighted graph, and indeed the formula reduces to $\rho (G_{3}^{1,1,1},K_{3})=30/90=1/3$ as expected.

## Appendix E. Non-neutral mutants

To illustrate the robustness of our results, in figure 11 we present the analogue of figure 4 for the case when mutants have a fitness advantage *r* ∈ {1.1, 1.5, 2}. The results are qualitatively similar to the neutral case *r* = 1.
